# Maine Council on Aging: Empowering Change Agents

**DOI:** 10.1093/geroni/igaf122.353

**Published:** 2025-12-31

**Authors:** Jess Mauer

**Affiliations:** Maine Council on Aging, Brunswick, Maine, United States

## Abstract

The Maine Council on Aging (MCOA) is a broad, multidisciplinary network of more than 135 organizations, businesses, municipalities, and community members working to ensure we can all live healthy, engaged, and secure lives as we age in our homes and community settings. The Power in Aging Project is a campaign to dismantle ageism and build an Age-Positive Maine by 2032. The Project’s primary strategy is to engage audiences from different sectors in direct conversations about age- bias, the impacts of ageism, and the benefits of living and working in an age-positive culture. Last year we achieved a historic victory that will increase the financial security of more than 45,000 older Mainers. These Mainers are now eligible for the Medicare Savings Program, a federal health insurance program that pays their Medicare costs. They will save on average $7,300 every year for the rest of their lives. We also celebrate our partnership with Hannaford Supermarkets and the $1.3 million grant they gave us to increase the food security and social connection of older people in our region, prioritizing diverse older people, older people living rurally, and veterans. By completion of the project, MCOA will have helped Hannaford make over $800,000 in investments in sustainable programs. Another exciting program was a relaunched Creative Aging Program that will be facilitating and hosting conversations and using creative processes to engage older people in discussions about their experiences with aging in Maine. In all our outreach data helps inform our work.

